# Expression of Serum Adenosine Deaminase in Pediatric Non-Hodgkin Lymphoma and Its Association with Clinical Outcomes and Survival

**DOI:** 10.3390/curroncol33030134

**Published:** 2026-02-25

**Authors:** Xiuli Zhu, Yuqiao Diao, Yan Chen

**Affiliations:** Department of Pediatrics, The Fourth Hospital of Hebei Medical University, Shijiazhuang 050011, China47300277@hebmu.edu.cn (Y.D.)

**Keywords:** adenosine deaminase, pediatric, Non-Hodgkin Lymphoma, clinical outcomes, survival

## Abstract

Background: Pediatric non-Hodgkin lymphoma (NHL) is a heterogeneous malignancy with variable outcomes. Adenosine deaminase (ADA), a key enzyme in purine metabolism, has been implicated in tumor progression and immune evasion, yet its role in pediatric NHL remains underexplored. Methods: This retrospective study included 215 pediatric NHL patients categorized into precursor cell lymphoma (n = 88) and mature cell lymphoma (n = 127) groups based on pathology. Patients were further defined into good (n = 143) and poor prognosis (n = 72) groups according to international response criteria. Serum ADA and other laboratory parameters were measured at diagnosis. Results: Precursor cell lymphomas showed higher rates of bone marrow involvement, peripheral blood involvement, and clinical stage IV disease compared to mature cell lymphomas. ADA levels were significantly elevated in precursor cell lymphomas and in the poor prognosis group. Elevated ADA was strongly correlated with a poor prognosis. Multivariable analysis identified precursor cell lymphoma, fever, bone marrow involvement, elevated LDH, and elevated ADA as independent predictors of poor prognosis (all *p* < 0.05). Conclusions: Serum ADA is significantly elevated in pediatric NHL, particularly in precursor cell subtypes and poor prognosis cases, and serves as a potential prognostic marker. ADA may help improve risk stratification and guide personalized treatment strategies.

## Simple Summary

Childhood non-Hodgkin lymphoma is a serious cancer where some children respond well to treatment while others do not. Doctors need simple blood tests to identify high-risk patients early. Our research focused on a blood enzyme called adenosine deaminase (ADA). We measured ADA levels in 215 children with lymphoma at the time of their diagnosis. We found that children with more aggressive types of lymphoma, especially those whose cancer cells were less mature, had much higher ADA levels in their blood. Importantly, children with high ADA levels were significantly more likely to have a poor response to standard chemotherapy. Our study suggests that a routine blood test for ADA could help doctors better predict which children have high-risk disease. This may allow for earlier treatment adjustments and closer monitoring, potentially improving care for these patients. Further studies are needed to confirm these findings.

## 1. Introduction

Non-Hodgkin lymphoma (NHL) represents a heterogeneous group of malignancies originating from lymphoid tissues, with variability in clinical presentation, biological behavior, and response to therapy [1]. Among pediatric populations, NHL constitutes a substantial proportion of childhood cancers, presenting unique challenges due to its aggressive nature and rapid progression [2]. Early diagnosis and effective treatment are critical for improving survival rates and minimizing long-term complications [3]. However, despite advancements in therapeutic strategies, identifying reliable biomarkers that can predict disease severity and guide personalized treatment remains an unmet need.

Adenosine deaminase (ADA), an enzyme involved in purine metabolism, has garnered attention for its role in immune regulation and inflammation. In the context of cancer, ADA activity has been linked to tumor proliferation, metastasis, and immune evasion [4]. Previous studies have demonstrated elevated ADA levels in various malignancies, including adult NHL, suggesting its potential as a biomarker for disease aggressiveness [5,6]. Limited data exist regarding the expression and clinical significance of ADA in pediatric NHL, highlighting the need for further investigation.

The pathophysiology of NHL involves complex interactions between genetic mutations, immune dysregulation, and metabolic alterations. Precursor cell lymphomas, characterized by the malignant transformation of immature lymphocytes, often exhibit rapid growth and high rates of systemic dissemination. These tumors are associated with distinct clinical features and outcomes compared to mature cell lymphomas, which include diffuse large B-cell lymphoma, Burkitt lymphoma, and others [7,8]. Understanding the molecular and metabolic underpinnings of these subtypes is crucial for developing targeted therapies and improving patient outcomes. Metabolic enzymes such as ADA may play a pivotal role in this context, influencing tumor biology through their effects on nucleotide metabolism and immune modulation.

Immune evasion is a hallmark of many cancers, including NHL, where tumor cells exploit various mechanisms to escape immune surveillance. ADA’s involvement in purine metabolism not only supports cellular proliferation but also modulates the tumor microenvironment, promoting immune tolerance and facilitating tumor growth [9,10]. In pediatric NHL, where the immune system is still developing, understanding how elevated ADA is associated with an immunosuppressive microenvironment could provide insights into novel therapeutic targets. Moreover, ADA’s role in regulating adenosine levels within the tumor microenvironment suggests its potential as both a prognostic marker and a therapeutic target. Adenosine, a downstream product of ADA activity, has immunosuppressive properties that can inhibit anti-tumor immune responses, thereby contributing to disease progression [11,12].

Given the complexity of NHL, integrating multiple biomarkers into clinical practice offers a promising approach for enhancing diagnostic accuracy and guiding personalized treatment. Serum ADA levels, along with other established markers such as lactate dehydrogenase (LDH), may provide complementary information for risk stratification and prognosis. LDH, a well-known marker of cellular turnover and stress, has been extensively studied in NHL and shown to correlate with disease burden and outcome [13,14]. Hydroxybutyrate dehydrogenase (HBDH), an isoenzyme of LDH, has recently emerged as a promising and independent prognostic biomarker in B-cell lymphomas, where its elevated levels are strongly associated with poorer survival and distinct metabolic pathways [15]. The combination of ADA and LDH could offer a more comprehensive assessment of disease activity and prognosis, potentially improving patient management. Exploring the interplay between these markers and other clinical parameters may reveal new avenues for therapeutic intervention.

By investigating the association between ADA levels and clinical outcomes, this study aims to contribute to the growing body of knowledge on the utility of metabolic enzymes as biomarkers in pediatric NHL. Such efforts will be instrumental in developing more effective treatment strategies and ultimately enhancing the care of children with NHL.

## 2. Materials and Methods

### 2.1. Study Population and Ethical Considerations

This single-center, retrospective cohort study was conducted after obtaining approval from the Institutional Review Board and Ethics Committee of the Fourth Hospital of Hebei Medical University (Approval Number: 2023KS142). The requirement for individual informed consent was waived by the Ethics Committee due to the retrospective nature of the study and the use of exclusively de-identified patient data and archived biological samples. This waiver is in accordance with national regulations and institutional guidelines for retrospective biomedical research. All patients or their guardians had provided broad consent for the use of residual clinical samples for future research at the time of hospitalization, as per the hospital’s standard procedure.

We screened the medical records of pediatric patients with newly diagnosed NHL who were admitted to our hospital between January 2016 and August 2022. A total of 215 patients who met the following criteria were included in the final analysis:

Inclusion Criteria: ① diagnosis of NHL confirmed by histopathological examination; ② availability of complete clinical staging data; ③ availability of complete baseline demographic, clinical, and laboratory data; ④ aged ≤ 14 years. Exclusion Criteria: ① lack of definitive pathological diagnosis or clinical staging; ② incomplete medical records or missing essential data; ③ diagnosis of a second primary malignancy; ④ patients with a prior history of chemotherapy or radiotherapy; ⑤ patients who were lost to follow-up.

### 2.2. Group Allocation, Data Collection, and Efficacy Evaluation

Eligible patients were initially categorized into two groups based on their pathological diagnosis: the precursor cell lymphoma group (n = 88), which included T-lymphoblastic and B-lymphoblastic lymphoma, and the mature cell lymphoma group (n = 127), which included diffuse large B-cell lymphoma, Burkitt lymphoma, anaplastic large-cell lymphoma, and extranodal NK/T-cell lymphoma. The grouping of mature cell lymphomas was based on the shared conceptual framework of the mature phenotype, which is fundamentally distinct from the ‘precursor’ lineage in clinical presentation and treatment paradigms in pediatric NHL. This dichotomy serves as a primary diagnostic axis, and the primary aim was to evaluate ADA across this major pathological divide. Demographic information, clinical characteristics, and laboratory data at initial diagnosis were systematically collected from the electronic medical record system.

Night sweats were defined as profuse sweating, resulting in drenched bedclothes. Bone marrow involvement was defined as the presence of ≥5% lymphoblasts in bone marrow aspirate by morphology or the detection of malignant cells by flow cytometry [16]. Central nervous system (CNS) involvement was diagnosed based on the CSCO Lymphoma Guidelines 2021 [16], defined as CNS2 (pleocytosis with blasts) or CNS3 (presence of blasts or cranial nerve palsy) in cerebrospinal fluid (CSF) analysis by cytospin and flow cytometry, or evidence of intracranial involvement on imaging. Clinical Staging was performed according to the St. Jude NHL Staging System [17]. Stage IV disease was defined as the presence of CNS involvement and/or bone marrow involvement. Bulky mass was defined as a tumor mass with a single diameter ≥10 cm on computed tomography (CT). For mediastinal masses, it was defined as a mass with a maximum transverse diameter greater than one-third of the internal thoracic diameter or >35% of the thoracic internal diameter at the T5–T6 level.

Patient outcomes were assessed based on the 2-year follow-up evaluation. The entire cohort was subsequently re-categorized into two prognostic groups according to the International Pediatric NHL Response Criteria [18]: The good prognosis group (n = 143) included patients who achieved complete response (CR) or partial response (PR) and maintained remission without relapse. The poor prognosis group (n = 72) included patients with stable disease (SD), progressive disease (PD), or those who experienced relapse after initially achieving chemotherapy-induced remission.

### 2.3. Blood Samples and Biochemical Assays

Fasting venous blood samples were collected from all subjects at the time of initial diagnosis. Serum was separated by centrifugation within 2 h of collection and aliquoted into cryovials. All serum samples were immediately frozen at −80 °C and stored in the hospital’s biobank until analysis.

Complete blood count, including white blood cell (WBC), red blood cell (RBC), hemoglobin (Hb), and platelet (PLT) counts, was performed using a fully automated hematology analyzer (Sysmex XN-9000, Sysmex Corporation, Kobe, Japan). LDH and hydroxybutyrate dehydrogenase (HBDH) levels were measured using an automated biochemical analyzer (Cobas c 501 module, Roche Diagnostics, Rotkreuz, Switzerland) with standardized ultraviolet-rate methods. Serum ADA activity was quantified using an automated biochemical analyzer (Cobas c 501 module, Roche Diagnostics, Rotkreuz, Switzerland) with a commercial reagent kit based on the adenosine deaminase assay principle. ADA activity was retrospectively measured from these archived frozen serum samples. Prior to ADA measurement, frozen serum samples were thawed at 4 °C and centrifuged to remove any precipitates. All samples were processed in a single batch to minimize inter-assay variability. All laboratory procedures were conducted in accordance with the manufacturer’s instructions and the standard operating procedures of the hospital’s central laboratory. The upper limit of normal (ULN) for serum ADA in our laboratory is 19.0 U/L, established from a local healthy pediatric population. This value was used to define elevated ADA for the analysis of abnormality rates presented.

### 2.4. Treatment Protocol

All pediatric NHL patients included in this study received risk-stratified, histology-specific chemotherapy protocols according to the contemporary Chinese Children’s Cancer Group (CCCG) or Children’s Oncology Group (COG) inspired guidelines, which were standardly applied at our institution during the study period (2016–2022). Treatment assignment was primarily based on the histopathological subtype and clinical stage at diagnosis, ensuring uniformity within each diagnostic category.

For patients with precursor cell lymphomas (T-lymphoblastic and B-lymphoblastic lymphoma), the treatment backbone consisted of intensive, multi-agent chemotherapy regimens modeled after the Berlin–Frankfurt–Münster (BFM) protocols for acute lymphoblastic leukemia. This typically included induction, consolidation, and maintenance phases with CNS prophylaxis.

For patients with mature cell lymphomas (including diffuse large B-cell lymphoma, Burkitt lymphoma, anaplastic large-cell lymphoma, and extranodal NK/T-cell lymphoma), short-term, high-intensity chemotherapy protocols were used. The specific regimen (e.g., LMB-based for high-grade B-cell lymphomas, ALCL99-based for anaplastic large-cell lymphoma) was selected based on the precise pathological diagnosis and stage.

Dose reductions or delays were strictly documented and were primarily due to treatment-related toxicities (e.g., myelosuppression, infection) rather than arbitrary protocol deviations. Supportive care measures were standardized.

Given that treatment was protocol-driven and highly consistent within each of the two major pathological groups (precursor vs. mature cell lymphoma), it was not a source of significant outcome variation within these categories for the purpose of this biomarker study. Therefore, the treatment protocol was not included as a separate variable in the multivariate Cox regression analysis, which focused on identifying baseline clinical and laboratory prognostic factors.

### 2.5. Statistical Analysis

Statistical analysis was performed using SPSS Statistics version 29.0 (IBM Corp., Armonk, NY, USA). Categorical variables are presented as frequencies and percentages [n (%)] and were compared using the chi-square test or Fisher’s exact test, as appropriate. Continuous variables were tested for normality using the Shapiro–Wilk test. Normally distributed data are expressed as the mean ± standard deviation (Mean ± SD) and were compared using the independent-sample *t*-test. Correlations between prognostic factors were assessed using Spearman correlation analysis. To identify independent prognostic factors, a multivariable Cox proportional hazards regression analysis was performed. Results are presented as hazard ratios (HR) with 95% confidence intervals (CIs). To further evaluate the prognostic value of serum ADA, event-free survival (EFS) was analyzed using the Kaplan–Meier method. Event-free survival (EFS) was defined as the time from diagnosis to the first occurrence of any of the following events: disease progression, relapse, or death from any cause. Patients who did not experience any of these events were censored at the date of their last follow-up. Patients were stratified into high- and low-ADA groups based on the optimal cut-off value of 25.9 U/L derived from the receiver operating characteristic (ROC) curve analysis for predicting poor prognosis. Differences between survival curves were compared using the log-rank test. A two-tailed *p*-value of <0.05 was considered statistically significant for all analyses.

## 3. Results

### 3.1. Expression of Serum Adenosine Deaminase in Pediatric Non-Hodgkin Lymphoma

The comparison of baseline demographics between the precursor cell lymphoma group and the mature cell lymphoma group (Table 1) revealed no significant differences in age, gender distribution, or BMI (all *p* > 0.05). Similarly, there were no significant differences in age distribution across different age categories (*p* > 0.05). Significant differences were observed in the primary site or type of lymphoma (*χ*^2^ = 61.9, *p* < 0.001). The precursor cell lymphoma group had a higher proportion of cases involving the thorax (50.00% vs. 10.24%) and a lower proportion involving the abdomen or retroperitoneum (10.23% vs. 50.39%) compared to the mature cell lymphoma group. Regarding pathological diagnosis, the precursor cell lymphoma group consisted of T-lymphoblastic lymphoma (64 cases) and B-lymphoblastic lymphoma (24 cases), while the mature cell lymphoma group included diffuse large B-cell lymphoma (26 cases), Burkitt lymphoma (50 cases), anaplastic large-cell lymphoma (46 cases), and extranodal NK/T-cell lymphoma, nasal type (5 cases).

The comparison of clinical characteristics between the precursor cell lymphoma group and the mature cell lymphoma group (Table 2) showed no significant differences in splenomegaly, CNS involvement, or EBV infection (all *p* > 0.05). Significant differences were noted for bone marrow involvement (70.45% vs. 16.54%, *χ*^2^ = 63.8, *p* < 0.001), peripheral blood involvement (62.5% vs. 9.45%, *χ*^2^ = 68.2, *p* < 0.001), and clinical stage IV (70.45% vs. 20.47%, *χ*^2^ = 53.7, *p* < 0.001).

**Figure 1:**
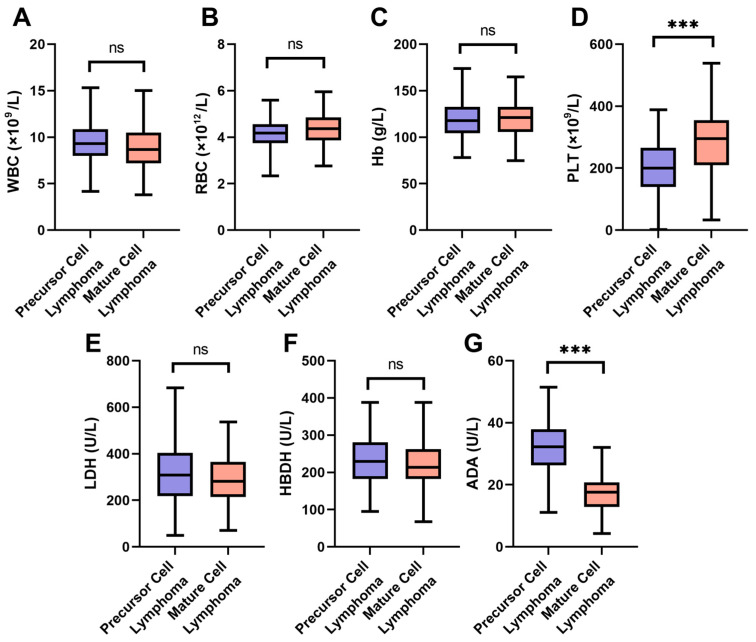
Comparison of laboratory test indicators between the two groups: (**A**) WBC; (**B**) RBC; (**C**) Hb; (**D**) PLT; (**E**) LDH; (**F**) HBDH; (**G**) ADA. ns: no significant difference; ***: *p* < 0.001. WBC: white blood cell; RBC: red blood cell; Hb: hemoglobin; PLT: platelet; LDH: lactate dehydrogenase; HBDH: hydroxybutyrate dehydrogenase; ADA: adenosine deaminase.

As shown in Figure 1, comparing laboratory test indicators between the precursor cell lymphoma group and the mature cell lymphoma group, no significant differences were found in WBC, RBC, Hb, LDH, or HBDH (all *p* > 0.05). PLT was significantly lower in the precursor cell lymphoma group (198.34 ± 91.57 vs. 287.63 ± 97.26, *t* = 6.78, *p* < 0.001), and ADA levels were significantly higher (32.36 ± 8.74 vs. 16.85 ± 5.12, *t* = 14.97, *p* < 0.001).

In comparing the abnormality rates of laboratory test indicators between the precursor cell lymphoma group and the mature cell lymphoma group (Table 3), no significant differences were found in WBC, RBC, Hb, LDH, or HBDH (all *p* > 0.05). PLT abnormalities were more frequent in the precursor cell lymphoma group (43.18% vs. 22.83%, *χ*^2^ = 10.0, *p* = 0.002), and ADA abnormalities were markedly higher (68.18% vs. 17.32%, *χ*^2^ = 57.0, *p* < 0.001).

### 3.2. Association of Serum Adenosine Deaminase with Clinical Outcomes and Survival in Pediatric Non-Hodgkin Lymphoma

The comparison of baseline demographics between the good prognosis group and the poor prognosis group (Table 4) revealed no significant differences in age, BMI, gender distribution, splenomegaly, or EBV infection (all *p* > 0.05). The proportion of patients with precursor cell lymphoma was significantly lower in the good prognosis group compared to the poor prognosis group (27.97% vs. 66.67%, *χ*^2^ = 29.7, *p* < 0.001). The presence of a bulky mass was also more common in the poor prognosis group (11.19% vs. 27.78%, *χ*^2^ = 9.45, *p* = 0.002). Fever was reported more frequently in the poor prognosis group (14.69% vs. 31.94%, *χ*^2^ = 8.76, *p* = 0.003). Additionally, bone marrow involvement (31.47% vs. 51.39%, *χ*^2^ = 8.05, *p* = 0.005) and peripheral blood involvement (25.17% vs. 43.06%, *χ*^2^ = 7.14, *p* = 0.008) were both more prevalent in the poor prognosis group.

**Figure 2:**
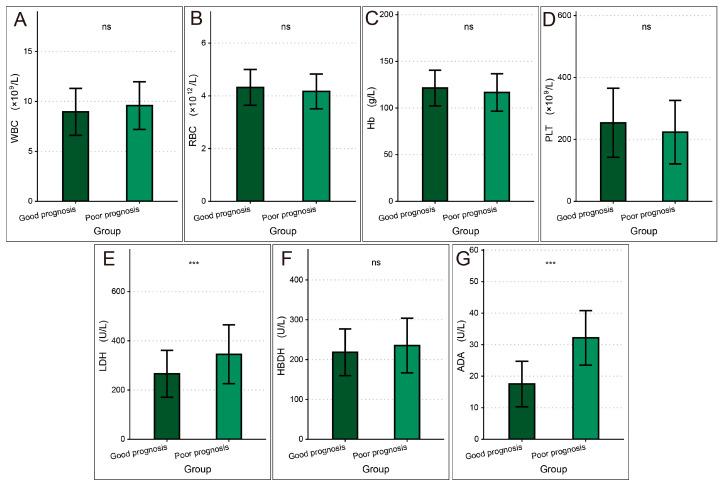
Comparison of laboratory and biochemical markers between the two groups: (**A**) WBC; (**B**) RBC; (**C**) Hb; (**D**) PLT; (**E**) LDH; (**F**) HBDH; (**G**) ADA. ns: no significant difference; ***: *p* < 0.001. WBC: white blood cell; RBC: red blood cell; Hb: hemoglobin; PLT: platelet; LDH: lactate dehydrogenase; HBDH: hydroxybutyrate dehydrogenase; ADA: adenosine deaminase.

In comparing laboratory and biochemical markers between the good prognosis group and the poor prognosis group (Figure 2), no significant differences were found in WBC, RBC, Hb, PLT, or HBDH (all *p* > 0.05). LDH was significantly higher in the poor prognosis group (345.28 ± 119.6 vs. 265.85 ± 95.34, *t* = 4.91, *p* < 0.001), as was ADA (32.15 ± 8.65 vs. 17.54 ± 7.21, *t* = 13.10, *p* < 0.001).

The correlation analysis of prognostic factors in pediatric non-Hodgkin lymphoma (Table 5) revealed significant correlations between several parameters and prognosis. ADA showed the strongest correlation (r = 0.662, *p* < 0.001), followed by LDH (r = 0.316, *p* < 0.001) and precursor cell lymphoma status (r = 0.371, *p* < 0.001). Bulky mass (r = 0.210, *p* = 0.002), fever (r = 0.202, *p* = 0.003), bone marrow involvement (r = 0.194, *p* = 0.004), and peripheral blood involvement (r = 0.182, *p* = 0.007) also showed significant, albeit weaker, correlations with prognosis.

**Figure 3:**
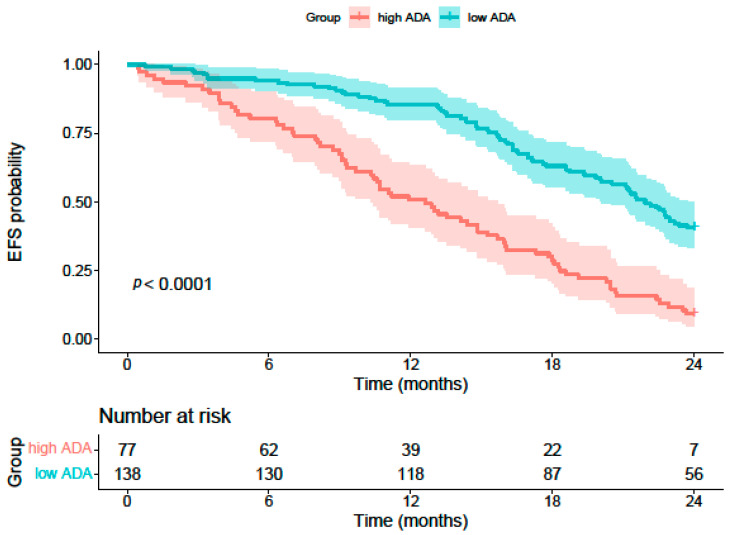
Kaplan–Meier curves for event-free survival in pediatric NHL patients stratified by serum ADA levels using the cut-off of 25.9 U/L (high ADA, n = 77 vs. low ADA, n = 138).

The multivariable Cox regression analysis (Table 6) identified precursor cell lymphoma (*p* < 0.001, HR = 2.15, 95% CI: 1.14–4.06), fever (*p* = 0.040, HR = 1.32, 95% CI: 1.25–2.20), bone marrow involvement (*p* = 0.013, HR = 1.82, 95% CI: 1.04–3.21), LDH (*p* = 0.005, HR = 1.02, 95% CI: 1.01–1.06), and ADA (*p* < 0.001, HR = 2.45, 95% CI: 1.86–3.26) as significant risk factors for a poor prognosis in pediatric non-Hodgkin lymphoma. Bulky mass (*p* = 0.109) and peripheral blood involvement (*p* = 0.432) were not significantly associated with prognosis (both *p* > 0.05). Kaplan–Meier analysis demonstrated significantly worse EFS in patients with high serum ADA levels compared to those with low levels (log-rank *p* < 0.0001; Figure 3). By the end of the 24-month follow-up period, only 7 patients in the high-ADA group remained at risk without experiencing an event, compared to 56 patients in the low-ADA group.

## 4. Discussion

The present study aimed to investigate the expression of serum ADA in pediatric NHL and its association with clinical outcomes and survival. Our findings highlight several key observations that contribute to a deeper understanding of the role of ADA in this context. We noted differences in primary site involvement and clinical characteristics between precursor cell lymphoma and mature cell lymphoma groups. Precursor cell lymphomas were more frequently associated with thoracic involvement and less often with abdominal or retroperitoneal sites compared to mature cell lymphomas. This anatomical distribution may reflect underlying biological differences in tumor biology and growth patterns between these subtypes [19,20].

Bone marrow and peripheral blood involvement were more prevalent in precursor cell lymphomas than in mature cell lymphomas. These findings suggest that precursor cell lymphomas may have a greater propensity for systemic dissemination at diagnosis. The higher rate of bone marrow involvement in precursor cell lymphomas could be attributed to their aggressive nature and rapid proliferation rates [21,22]. This clinical pattern is consistent with the recognized biological continuum between lymphoblastic leukemia and lymphoma, where the distinction is primarily based on the extent of bone marrow involvement at presentation [23]. Understanding these patterns can help guide initial diagnostic workup and risk stratification strategies [24,25].

Serum ADA levels were elevated in patients with precursor cell lymphoma compared to those with mature cell lymphoma. Adenosine deaminase plays a critical role in purine metabolism, particularly in immune regulation and inflammation. In cancer settings, increased ADA activity has been linked to enhanced cellular proliferation and immune evasion. It is plausible that the higher ADA levels observed in precursor cell lymphomas reflect heightened metabolic activity and immune modulation within these tumors [12,26]. Further studies are needed to elucidate the exact mechanisms by which ADA contributes to lymphomagenesis and disease progression.

In terms of clinical outcomes, precursor cell lymphomas were more frequently associated with a poor prognosis compared to mature cell lymphomas. Patients in the poor prognosis group exhibited higher ADA levels, along with other adverse prognostic factors such as bulky mass, fever, and bone marrow involvement. Elevated ADA levels in the poor prognosis group suggest that ADA may serve as a biomarker for more aggressive disease and poorer clinical outcomes. ADA’s role in modulating the tumor microenvironment and promoting immune escape could explain its association with a worse prognosis. Additionally, ADA’s involvement in nucleotide metabolism might support tumor growth and resistance to therapy. Future research should focus on exploring these mechanisms further to identify potential therapeutic targets [27,28,29].

LDH levels were also higher in the poor prognosis group, indicating that LDH and ADA may act synergistically to influence disease progression. Both enzymes play crucial roles in cellular metabolism and stress responses, potentially contributing to the aggressive behavior of certain lymphomas [30,31]. The correlation between ADA levels and clinical outcomes suggests that ADA could be integrated into existing prognostic models to improve risk stratification. It is important to note that while ADA shows promise as a biomarker, its utility needs to be validated in larger, prospective studies.

It is important to acknowledge that elevated serum ADA, like LDH, likely reflects high tumor burden and proliferative activity, which are hallmarks of aggressive lymphoma. The strong correlation observed between ADA and LDH supports this notion. Therefore, while our data intriguingly associate high ADA with poor outcomes and a precursor phenotype, they cannot distinguish whether ADA is a direct mediator of pathogenesis via purine metabolism modulation or primarily a robust surrogate marker of aggressive disease biology and turnover.

Multivariable Cox regression analysis identified several factors, including precursor cell lymphoma status, fever, bone marrow involvement, LDH, and ADA, as significant predictors of poor prognosis. These findings underscore the complexity of NHL pathophysiology and the need for multifaceted approaches to risk assessment and treatment planning. Precursor cell lymphomas, in particular, pose significant challenges due to their aggressive nature and high rates of systemic involvement. Identifying reliable biomarkers like ADA can aid in early detection and tailored therapeutic interventions, ultimately improving patient outcomes [32,33,34]. The standardized, pathology-driven treatment protocols applied to all patients minimize the confounding effect of heterogeneous therapies on the prognostic associations observed, strengthening the validity of our findings regarding ADA and other baseline risk factors.

Despite these insights, our study has several limitations. The retrospective design limits our ability to establish causality between ADA levels and clinical outcomes. The sample size, while adequate, may not be sufficiently large to detect smaller differences in treatment effects. Future studies should consider these factors to provide more robust evidence supporting the efficacy of ADA as a biomarker in pediatric NHL. Randomized controlled trials with larger sample sizes and longer follow-up periods are necessary to confirm the long-term benefits and safety of using ADA levels in clinical practice. Additionally, the mature cell lymphoma group amalgamates entities with diverse biologies (e.g., Burkitt lymphoma, DLBCL, ALCL). While this grouping allowed us to test ADA across the fundamental precursor–mature dichotomy, it may obscure subtype-specific variations in ADA expression. Future studies with larger cohorts of each subtype are warranted to explore such differences.

This study provides valuable insights into the role of serum ADA in pediatric NHL and its association with clinical outcomes and survival. Elevated ADA is associated with precursor cell lymphomas and poor prognosis, highlighting its potential as a biomarker for disease aggressiveness. Further research is warranted to validate these findings and explore the underlying mechanisms by which ADA influences lymphoma biology. By integrating ADA into clinical decision-making processes, we may enhance risk stratification and personalize treatment strategies, ultimately improving the care and outcomes for children with NHL. Future directions should include longitudinal studies to assess changes in ADA levels over time and their impact on treatment response and survival.

## 5. Conclusions

This study suggests that serum ADA levels may play a role in the pathophysiology of pediatric NHL, potentially serving as a biomarker for disease aggressiveness and prognosis. Elevated ADA levels were observed in patients with precursor cell lymphomas, which are associated with more aggressive clinical characteristics such as higher rates of bone marrow involvement and systemic dissemination. Furthermore, ADA was linked to poorer clinical outcomes, indicating its potential utility in identifying patients at higher risk of adverse events. This research underscores the need for continued investigation into the role of ADA and other metabolic enzymes in pediatric NHL, aiming to enhance our understanding and management of this complex disease.

## Figures and Tables

**Table 1:** Comparison of baseline demographics between the precursor cell lymphoma and mature cell lymphoma groups.

Parameters	Precursor Cell Lymphoma (n = 88)	Mature Cell Lymphoma (n = 127)	*t*/*χ*^2^	*p*
Age (years)	8.49 ± 2.20	8.71 ± 2.11	0.681	0.50
Distribution [n (%)]			0.028	0.999
<6 years	11 (12.50%)	15 (11.81%)		
6 to <12 years	57 (64.77%)	83 (65.35%)		
12 to <15 years	15 (17.05%)	22 (17.32%)		
≥15 years	5 (5.68%)	7 (5.51%)		
BMI (kg/m^2^)	14.95 ± 1.46	15.11 ± 1.38		
Gender [n (%)]			0.175	0.676
Male	66 (75.00%)	92 (72.44%)		
Female	22 (25.00%)	35 (27.56%)		
Primary site or type [n (%)]			61.9	<0.001
Thorax	44 (50.00%)	13 (10.24%)		
Abdomen or retroperitoneum	9 (10.23%)	64 (50.39%)		
Head and neck, except skin and nodes	13 (14.77%)	26 (20.47%)		
Peripheral lymph node	13 (14.77%)	20 (15.75%)		
Cerebral lymphoma	2 (2.27%)	2 (1.57%)		
Other tumor site	7 (7.95%)	2 (1.57%)		
Pathological diagnosis	T-lymphoblastic lymphoma: 64 cases; B-lymphoblastic lymphoma: 24 cases	Diffuse large B-cell lymphoma: 26 cases; Burkitt lymphoma: 50 cases; Anaplastic large-cell lymphoma: 46 cases; Extranodal NK/T-cell lymphoma, nasal type: 5 cases		

BMI: body mass index; NK: natural killer.

**Table 2:** Comparison of clinical characteristics between the two groups.

Parameters	Precursor Cell Lymphoma (n = 88)	Mature Cell Lymphoma (n = 127)	*χ* ^2^	*p*
Splenomegaly	20 (22.73%)	20 (15.75%)	1.67	0.196
Bone Marrow Involvement	62 (70.45%)	21 (16.54%)	63.8	<0.001
Peripheral Blood Involvement	55 (62.5%)	12 (9.45%)	68.2	<0.001
CNS Involvement	23 (26.14%)	22 (17.32%)	2.44	0.118
EBV Infection	13 (14.77%)	27 (21.26%)	1.45	0.229
Clinical Stage IV	62 (70.45%)	26 (20.47%)	53.7	<0.001

EBV: Epstein–Barr virus; CNS: central nervous system.

**Table 3:** Comparison of laboratory test indicators’ abnormality rates between the two groups.

Parameters	Precursor Cell Lymphoma (n = 88)	Mature Cell Lymphoma (n = 127)	*χ* ^2^	*p*
WBC [n (%)]	40 (45.45%)	48 (37.8%)	1.26	0.261
RBC [n (%)]	28 (31.82%)	37 (29.13%)	0.178	0.673
Hb [n (%)]	43 (48.86%)	61 (48.03%)	0.014	0.904
PLT [n (%)]	38 (43.18%)	29 (22.83%)	10.0	0.002
LDH [n (%)]	62 (70.45%)	73 (57.48%)	3.75	0.053
HBDH [n (%)]	47 (53.41%)	57 (44.88%)	1.51	0.219
ADA [n (%)]	60 (68.18%)	22 (17.32%)	57.0	<0.001

WBC: white blood cell; RBC: red blood cell; Hb: hemoglobin; PLT: platelet; LDH: lactate dehydrogenase; HBDH: hydroxybutyrate dehydrogenase; ADA: adenosine deaminase.

**Table 4:** Comparison of baseline demographics between the good prognosis and poor prognosis groups.

Parameters	Good Prognosis Group (n = 143)	Poor Prognosis Group (n = 72)	*t*/*χ*^2^	*p*
Age (years)	9.56 ± 3.06	9.62 ± 3.11	0.123	0.902
BMI (kg/m^2^)	15.22 ± 1.43	14.87 ± 1.45	1.68	0.095
Gender [n (%)]			1.64	0.200
Male	109 (76.22%)	49 (68.06%)		
Female	34 (23.78%)	23 (31.94%)		
Precursor Cell Lymphoma/Mature Cell Lymphoma [n (%)]	40 (27.97%)	48 (66.67%)	29.7	<0.001
Bulky Mass [n (%)]	16 (11.19%)	20 (27.78%)	9.45	0.002
Fever [n (%)]	21 (14.69%)	23 (31.94%)	8.76	0.003
Splenomegaly [n (%)]	25 (17.48%)	15 (20.83%)	0.355	0.551
Bone Marrow Involvement [n (%)]	45 (31.47%)	37 (51.39%)	8.05	0.005
Peripheral Blood Involvement [n (%)]	36 (25.17%)	31 (43.06%)	7.14	0.008
EBV Infection [n (%)]	27 (18.88%)	17 (23.61%)	0.658	0.417

EBV: Epstein–Barr virus; BMI: Body mass index.

**Table 5:** Correlation analysis of prognostic factors in pediatric non-Hodgkin lymphoma.

Parameters	r	*p*
Precursor Cell Lymphoma	0.371	<0.001
Bulky Mass	0.210	0.002
Fever	0.202	0.003
Bone Marrow Involvement	0.194	0.004
Peripheral Blood Involvement	0.182	0.007
LDH	0.316	<0.001
ADA	0.662	<0.001

LDH: lactate dehydrogenase; ADA: adenosine deaminase.

**Table 6:** Multivariable Cox regression analysis of prognostic factors in pediatric non-Hodgkin lymphoma.

Parameters	*p*	HR	95% CI
Precursor Cell Lymphoma	<0.001	2.15	1.14–4.06
Bulky Mass	0.109	1.49	0.856–2.56
Fever	0.040	1.32	1.25–2.20
Bone Marrow Involvement	0.013	1.82	1.04–3.21
Peripheral Blood Involvement	0.432	1.65	0.907–2.99
LDH	0.005	1.02	1.01–1.06
ADA	<0.001	2.45	1.86–3.26

LDH: lactate dehydrogenase; ADA: adenosine deaminase.

## Data Availability

The datasets used during the present study are available from the corresponding author upon reasonable request.
